# Habit, medicine, and society in 18th-century Britain

**DOI:** 10.1017/mdh.2025.10042

**Published:** 2026-01

**Authors:** Alexander Wragge-Morley

**Affiliations:** School of Global Affairs, Lancaster University, Lancaster, United Kingdom

**Keywords:** Habit, Medicine, Custom, Menstruation, Cullen, 18th Century

## Abstract

This article examines the place of habit in the medical thought and practices of 18th-century Britain. Scholars, including Steven Shapin and Phil Withington, have shown that habit was important to the broadly humoral understandings of health, disease, and regimen that dominated in Europe for much of the early modern period. In this article, I offer the first sustained attempt to understand the role of habit in the medical thought of 18th-century Britain, focusing on the influential Scottish physician William Cullen. For the first time engaging with all of Cullen’s work on habit, including his correspondence, pathological lectures, and clinical lectures, I show that medics of the 18th century developed a new understanding of habit, linked to changing ideas about the nervous system. Increasingly, they emphasised the role that habit could play in causing the periodical return of bodily functions, even when there appeared to be no plausible physical cause. In so doing, medics engaged with one of the key debates of the 18th century – the contested notion that human nature itself might be contingent on social and environmental conditions. For them, habit provided the means by which society could quite literally change the body. These ideas come through clearly in the striking suggestion – hitherto unnoticed – that menstruation was the product of habit, arising not from nature but from culture. Discussions of menstruation reveal the political stakes of habit, with links to highly contested debates about the role that bodies of different genders might play in society.

## Introduction

Habit is a fundamental part of experience. Habit gives humans the capacity to change their natures, using repetition to slowly turn voluntary actions into involuntary responses that require little thought or deliberation. But it is also a principle of continuity, with deeply ingrained habits frequently resisting our willed efforts to overcome them. An obvious and frequently cited example of this combined tendency towards change and continuity is what we now call addiction. The act of consuming alcoholic drinks, if repeated often enough, may transform what had once been a choice into a compulsion. Once established, the involuntary desire for alcohol can be hard to overcome, thwarting our efforts to re-establish voluntary control over our actions.

It was only around the turn of the 19th century, however, that medics in Europe and North America began to use the language of addiction to describe the effects of habit on mind and body. For most of the early modern period, as Phil Withington has shown, the dominant languages for describing such shifts from voluntary to involuntary action were those of habit and custom.^1^ This was because most medics of early modern Europe practised a broadly humoral kind of dietetic medicine that emphasized the effects of regimen – including food, exercise, climate, and the other so-called non-naturals - on an individual’s temperament. On this understanding, temperament was a semi-permanent disposition that could be altered by repeated exposure to different activities, consumption, and stimuli.^2^ As Steven Shapin has shown, this broadly humoral model of dietetics enabled doctors and patients to think about the effects of habit in physical terms. Medical theorists of the 16th and 17th centuries held that people could, for better or worse, use repeated patterns of activity and consumption to alter their temperaments – to give themselves new dispositions or tendencies that would in turn require distinctive medical treatments. It was therefore no exaggeration when, as Shapin points out, physicians claimed that habit was a ‘second nature’. Those physicians meant that habits quite literally altered the temperament, giving individuals a nature that supplanted the one with which they had been born.^3^

Despite these scholarly interventions, however, there has so far been little recognition that theorists and practitioners of medicine took a close interest in habit and its effects on the body. To the limited extent that scholars have explicitly thematized habit, they have characterized it as a manifestation of the early modern emphasis on dietetics and regimen. This characterization is accurate, but insufficient. Shapin has shown that medics of the 16th and 17th centuries saw the cultivation of habits as an important tool either for maintaining a healthy temperament or modifying an unhealthy one. For the moment, however, we are in the dark about what happened when medics adapted such long-standing ideas to the new explanatory frameworks that emerged during the 17th and 18th centuries. Although humoralism continued to exert a powerful influence, medics increasingly adopted models based on the agency of the nervous system – rather than the humours or the blood – to explain the causes of voluntary and involuntary motion, as well as the fluctuating relationships between them. One of the tasks of this essay, therefore, will be to look for patterns of continuity and change by finding out what happened to ideas about habit in the 18th century.

I will undertake this task by focusing on the extensive writings of the influential Scottish physician and university lecturer William Cullen (1710–1790). Cullen was one of the leading medics of his generation, teaching the theory and practice of medicine at the University of Edinburgh from 1756 until just before his death. During his lifetime, Cullen exerted a remarkably powerful influence through his charismatic and thoughtful teaching – an influence expressed by the many students who either reproduced or elaborated upon his ideas and methods. Although he published widely on medical topics, the lecture notes carefully preserved by students form a key resource for understanding Cullen’s thought.^4^ At the same time, he ran a thriving medical practice, treating the wealthy privately and often by correspondence, as well as tending to the poor at the Royal Infirmary of Edinburgh. As a result, Cullen’s voluminous clinical lectures and case notes, as well as correspondence, provide us with valuable insights into the ways he put his theories into practice.^5^ In all these areas of his teaching and practice, Cullen devoted considerable attention to habit. In his medical lectures, he addressed habit as a topic in its own right, as well as frequently invoking it in his discussions of specific diseases and bodily states. The same goes for his correspondence and clinical lectures, where Cullen identified habit as an important consideration in the causes and cures of disease.^6^

As well as being the leading medic of his generation, Cullen was a key figure in the Scottish Enlightenment, making connections with key intellectuals first in Glasgow, and then in Edinburgh when he moved there in 1756. He counted such luminaries as David Hume, Adam Smith, and Henry Home, Lord Kames, among his friends.^7^ Moreover, Cullen engaged explicitly with ongoing philosophical debates about the moral ramifications of habit in his medical thought, both for individuals and for whole societies. In fields as diverse as aesthetics, medicine, philosophy, and theology, habit had emerged as a means of accounting for shifting configurations of willed and unwilled action. Perhaps the most influential treatment – and one that would shape Hume’s treatment of habit – came from the English bishop Joseph Butler (1692–1752). In the 1730s, Butler had used habit to account for two seemingly contradictory tendencies. On the one hand, habit seemed to distance people from their actions. Through repetition, even virtuous actions may take on a species of automaticity, emerging regardless of our intentions. On the other hand, it was precisely this tendency that made it possible to *intentionally* embed virtuous intentions into our everyday lives. Through the initially arduous repetition of virtuous actions, individuals could take on a second nature – a nature that involuntarily expressed the virtuous foundations upon which it had been built. Here, Butler gave new voice to the claims of a long theological tradition, stretching back to medieval scholasticism and its ancient sources, that regarded the habitual expression of virtue as a good thing. Habit enabled people to encode new patterns of action into mind and body, replacing inborn instincts with new and potentially better ones. The cost, however, was that the self-same repetition bred a lack of voluntary control, with actions taking on an automatic quality that potentially emptied them of their ethical value.^8^

At the same time, philosophers mapped such ideas about voluntary and involuntary action onto their concerns about society. Habit figured prominently, for instance, in writing on education. Even though philosophers such as John Locke (1632–1704) and Cullen’s contemporary Thomas Reid (1710–1796) hoped that people might guide their actions through reason, they nevertheless recognised that in practice many actions are determined by entrenched habits. Since children began to acquire habits long before they could reason, it followed that habit determined many of their most fundamental mental and physical operations – and that the cultivation of good habits was one of the key tasks of education.^9^ In medicine, meanwhile, a resurgence of dietetic and environmental medicine, prompted in part by anxieties over the increasing availability of imported food and drink, led medics such as the fashionable diet doctor George Cheyne (1672–1743) to worry that new habits of consumption were producing new pathologies of mind and body.^10^ At the same time, European thinkers increasingly mobilised habit to explain what they perceived as different varieties of human nature, imagined to result from differences of climate, customs, diet, or political economy. Naturalists such as Georges-Louis Leclerc, Comte de Buffon (1707–1788), historians such as William Robertson (1721–1793), and philosophers like Jean-Jacques Rousseau (1712–1788) and Denis Diderot (1713–1784) all asserted that variations in climate and social organization produced distinctive varieties of human nature. They held that different forms of social organization, through the habits they engendered, could produce groups of humans who differed from each other, even at a biological level.^11^

Habit was therefore important to thinkers of the 18th century, giving them tools for discussing the relationships between individuals and society, as well as the possibility that human nature might change over time and place. The aim of this essay, however, is to show that habit also played a distinctive role in the medical thought and practices of that century. And in turn it will show that medical ideas about habit enable us to see precisely why some thinkers believed that environments and societies could alter the workings of mind and body. Whilst acknowledging that philosophy and medicine of the 18th century cannot be readily or neatly separated, this essay will dwell on the discussions of habit to be found in Cullen’s lectures, both from his own notes and those of his students, as well as a range of other published and archival sources left by him and his contemporaries. John P. Wright and Shuta Kiba have briefly discussed Cullen’s remarks on habit – remarks that appeared in sections dealing with ‘custom and habit’ in the many published and unpublished versions of his lectures.^12^ So far, however, nobody has examined the role that Cullen gave to habit in his ideas about the causes and treatment of specific diseases, scattered throughout his pathological and clinical lectures and correspondence. But it is precisely here, when relating habit to regularities and irregularities in the workings of the body, that Cullen and other medics made their most revealing claims. Through habit, they suggested, the workings of the human body be slowly transformed by a wide range of environmental and social factors.

As we shall see, there were many points of continuity between the broadly humoral model of habit dominant in the 16th and 17th centuries and ideas about habit among medics of the 18th century. But there were big changes too, and these changes are revealed most clearly if we attend to the specific diseases and bodily processes upon which medics of the 18th century tended to focus when they discussed habit. Among these were the phenomena of periodicity – the body’s tendency to acquire regular cycles of action, and for those cycles to persist even when the initial cause no longer seems to be present. These phenomena included the timing of those urges leading to ingestion and excretion, as well as sleep and waking.^13^ But they also included the periodical return of increased blood flow to different parts of the body, whether in menstruation, haemorrhoids, or by another passage. Indeed, Cullen was one of several medics and philosophers who regarded menstruation as a habit, taking place not through physical necessity, but instead because women’s bodies became accustomed to it. This surprising idea has so far received almost no attention from historians, whether in histories of habit or in histories of menstruation.^14^ We will see, however, that this interest in the periodicity of women’s bodies enables us to understand how medics and philosophers of the 18th century reckoned with the political stakes of habit. Medics such as Cullen embraced the notion that habit could be a liberating force, enabling patients to overcome diseases and even modify their temperaments. At the same time, however, they saw the exercise of this power as more appropriate for men than women. They suggested that people with feminine bodies might be ruled by habits imposed on them by social customs and environmental conditions – and even asserted that this form of domination would be appropriate.

The story of habit in the medical thought of the 18th century closely reflects, therefore, the political stakes attendant on discussions of habit today. The activist and sociologist Pierre Bourdieu famously used the term *habitus* – a term he found in the scholastic philosophy of the medieval period – to explain how people come to internalize the norms imposed by society. Although Bourdieu did not enter into biological or physiological speculation, he saw this process of internalization as a bodily one, memorably stating that ‘The social order inscribes itself in bodies.’^15^ More recently the sociologist Tony Bennett has reasserted this point, reminding us that the apparently thoughtless, repetitive quality of habits makes them powerful vehicles for domination. Through their habits, people may come to follow social conventions – even incorporate them into their bodies – without the least awareness that their actions reflect the interests of a political order.^16^ Today, scholars are beginning to explore what Tyler Leeds has called *bio-habitus* – the notion that the internalization of social norms may take place at the level of the body’s conditioned responses to external stimuli. Leeds, for instance, speculates that long-term exposure to pain may gradually alter an individual’s cognitive dispositions.^17^ In this essay, we will see that Cullen and his contemporaries also saw habit playing out at this biological level, identifying long-term changes in the body’s responses to its environment as the consequence of repeated exposure to social conditions. Moreover, they even speculated on the desirability of this biological conditioning, asserting that some people – people with feminine bodies – should be more subject to that conditioning than others.

## Habit in 18th-century medical thought

In his lectures at the University of Edinburgh on the theory of medicine for the years 1769–1770 and 1770–1771, William Cullen spoke at length about the effects of habit. Cullen argued that the formation of habits gave regular order to some of the body’s most basic functions. Among the phenomena he had in mind were the return of hunger at predictable times of day, along with the similarly predictable urge to defecate. Cullen’s point is remarkable both for its simplicity and for its wide application. He wanted to show that people do not become hungry simply because their stomachs are empty or defecate simply because the rectum is stimulated by faeces. Instead, we seem to develop the habit of feeling the sensations that govern those actions at regular times of day, regardless of the actual need. Somehow, people develop a kind of automaticity independent of physical necessity, determined not by the body’s immediate needs, but instead by habit.^18^ Much of what Cullen had to say in these lectures was consistent with the vision of habit deployed by medics of previous generations, working in the broadly humoral medical tradition characteristic of early modern Europe. Indeed, Cullen was in respects one of the last great exponents of this tradition, frequently conceptualising disease as a temperamental imbalance to be remedied – under his guidance – through individual attention to diet and regimen.^19^ Yet, Cullen’s interest in periodicity, and conceptualisation of its causes, also reflected major changes that took place during the 18th century, especially from the middle decades onwards.

Ideas about habit and custom had long been important to humoral medicine and retained considerable force throughout the 18th century too. It is worth noting from the outset that most medics and philosophers used the terms ‘habit’ and ‘custom’ interchangeably, obscuring the differences implicit in their derivation from the Latin terms *habitus* and *consuetudo.* The medieval philosopher Thomas Aquinas (ca. 1225–1274) had distinguished sharply between these two terms. He identified *consuetudo* or custom as something that humans had in common with other animals, with thoughtless repetition resulting in the acquisition of new capacities or dispositions. By contrast, he saw *habitus* as a capacity reserved for humans alone, characterizing it as the ability to internalize and thus enact virtues through a thoughtful, considered kind of repetition.^20^ We can perhaps see the faintest echoes of this distinction in the definitions of habit and custom put forward by both Cullen and the philosopher Henry Home, Lord Kames – definitions that would eventually preface their parallel articles on ‘Custom and Habit’ in the 1797 edition of *Encyclopædia Britannica.* They both identified custom as the repetition of an act, whereas habit was the effect of that repetition on mind and body. As they saw it, habit was the disposition arising from repetition.^21^ But even Cullen felt that this distinction was very fine, telling his students that ‘in general we can hardly distinguish’ the two senses of the word.^22^ Neither, it seems, could many other writers of the 18th century, who either picked just one of the terms or used the two as synonyms.^23^

In addition, as Shapin notes, early modern medical writers frequently used the term habit either as a synonym for a disposition to a given disease, or for an overall temperament – a pair of usages that persisted throughout the eighteenth century.^24^ In March 1784, for instance, a Dr. Smyth used the term in its dispositional sense when writing to Cullen about a patient referred to as Mrs R. Here, he remarked that Mrs R had a tendency to constipation by stating that ‘Her Habit of Body is costive.’ And later in the same letter he explained that the patient’s weak pulse left her at risk of developing a ‘Cachetic Habit’ – meaning that her body might become so weak as to develop symptoms such as oedematous swellings in the lower legs.^25^ But as well as using the term to refer to specific pathological dispositions, Cullen’s correspondents used it to refer to temperament – to disposition in its broader sense. In 1780, for instance, the doctor John Gilchrist opened a letter to Cullen by describing his patient Dr. Thomas Mutter as having ‘a corpulent and very sluggish habit’. Here, Gilchrist was referring to Mutter’s temperament in a general sense, characterising him as a man little disposed to exercise and with a tendency to put on weight. But in the same letter, Gilchrist also deployed the term to account for repeated acts or experiences going on in the body, explaining that Mutter was suffering from a ‘habitual’ malady – repeated diarrhoea. For Cullen and his contemporaries, in other words, the term habit referred both to the repeated acts experienced by individuals and to the semi-permanent dispositions that those acts produced.^26^

Given this tendency to think about habit in relation to long-standing ideas about temperament and disposition, much of what Cullen had to say about habit was essentially advice about regimen.^27^ He was sensitive, for example, to how the habitual consumption of intoxicants could alter the temperament, turning what had once been an occasional pleasure into a need that the body could no longer do without. In June 1776, he advised that John Crawford should continue drinking wine despite the risks it posed to his health, remarking that ‘his habits absolutely require the continuance of Wine’. Here, the aim was to coordinate the patient’s own sense of their body with the physician’s expertise in the management of the non-naturals. Under the doctor’s guidance, patients could use regimen to attain a state of health consistent with their temperament, or perhaps alter that temperament to render it less burdensome.^28^ We can see Cullen thinking in the same terms if we turn to his writings on the implications of habit for how people should look after their health. Mobilising long-standing authorities such as the ancient philosopher Celsus and the Venetian physician Santorio Santorio (1561–1636), he counselled a regime of moderate variety, arguing that such a way of life would prevent the acquisition of habits that might later prove prejudicial to health. But at the same time he insisted on the individuality of experience. For those with a weak constitution, he suggested, this regime would not do. Instead, following the advice of the famously long-lived Venetian nobleman Luigi Cornaro (d. 1566), such people should cultivate regular habits, lest small deviations damage their delicate bodies. Much of the time, Cullen therefore mobilised an approach based on a long history of dietetic medicine, premised on the idea that the management of the non-naturals could materially alter the temperament.^29^

By the second half of the 17th century, however, different explanatory possibilities emerged. The philosopher René Descartes (1596–1650) had argued that matter was inert and entirely passive, capable of moving only when moved by something else.^30^ The only thing that could actually cause motion was immaterial spirit – the substance out of which both God and the human soul were supposedly made. According to this strictly dualist distinction between matter and spirit, there were only two possible causes for the motions of the human body. The first was an act of will, deliberately chosen by the immaterial soul inhabiting the body. The second was physical necessity, taking place when the particles of matter making up the body were acted upon by other particles of matter – particles that had initially been set in motion at the beginning of time by God.^31^ Philosophers and medics of the 17th and 18th centuries found the implications of this account troubling. Although Descartes made the immaterial soul responsible for what we choose to do, this left physical necessity in charge of everything else – all the operations that take place involuntarily in the body, as well as, according to Descartes, every action undertaken by plants and animals. While medics and philosophers were often enthusiastic about Descartes’s model of physical causation, they nevertheless worried that it would make many of the body’s operations into products of blind necessity rather than wisdom and intentionality.^32^

During the later 17th and early 18th centuries, medics such as Claude Perrault (1613–1688) and Georg Ernst Stahl (1659–1734) responded to this problem by arguing that the immaterial soul did in fact direct all the body’s functions. To make this case, however, they had to overcome the objection that many of those operations seem to take place automatically, without the mind exercising voluntary control. How could the soul direct the body without being aware of what it was doing? For Perrault, habit provided a solution. At the beginning of their lives, he suggested, babies voluntary controlled all the basic functions of the body, such as digestion and the regulation of body temperature. Over time, however, habit made those operations recede from awareness, freeing the mind to focus on other things. The absence of awareness did not imply the absence of will or intelligence. Actions that seemed automatic or instinctive had originally been, and in some sense remained, willed actions ordained by an immaterial soul. Although Perrault provided the clearest articulation of these ideas, medics of the 18th century generally attributed them to Stahl – perhaps because Stahl exerted a broader influence through both his writings and his students.^33^

By the middle decades of the century, medics were less interested in the ontological questions that had been so important in the immediate wake of Descartes’s intervention. They remained preoccupied, however, by the notion that the body might express intentionality or intelligence in ways that defied straightforwardly material explanation – and Stahl’s ideas were a key source of inspiration for their efforts to reject straightforwardly mechanical explanations for bodily action. As Peter Hans Reill and others have shown, medics and physiologists active from the middle decades of the 18th century onwards were keenly interested in the notion that the matter of the body, through its organization into discrete structures, was capable of performing the functions necessary to life without the intervention of the mind. The Swiss physiologist Albrecht von Haller (1708–1777), for example, found that the muscles were irritable – that they appeared to respond to stimuli on their own, without either transmitting information to the mind or receiving instructions from it. This discovery, based on experimental evidence, led Haller to frame an influential distinction between what he called sensibility, and irritability. He used the term sensibility to describe sensations and motions of which the mind was aware, and irritability for those for which there was no perception.^34^ To be clear, the notion that the living body might on its own produce intentional or intelligent action contradicted Stahl’s position that the immaterial soul alone directed motion. Nevertheless, the idea that some parts of the body were irritable – that they had an innate capacity for motion – made it possible to talk about seemingly intelligent forms of motion in the body that did not have obvious physical causes.

During the middle decades of the 18th century, physicians in Scotland took up the challenge presented by these new models of bodily action. Consider, for instance, Robert Whytt (1714–1766), Cullen’s predecessor at Edinburgh and one of Haller’s key interlocutors.^35^ In his *An Essay on the Vital and other Involuntary Motions of Animals* (Edinburgh, 1751), Whytt intervened in a long-standing debate about the conjoint motion of the eyes. Like the ophthalmologist William Porterfield about two decades earlier, Whytt observed that there was no physical connection – no direct neurological connection – to explain why the eyes move together in unison to focus on the objects of vision. In the absence of an obvious material explanation, Whytt bought into Porterfield’s suggestion that this motion had something to do with habit. He argued that infants had at first voluntarily chosen to move their eyes together, motivated by the discomfort of having two different sources of visual information acting on the sensorium. But over time, and at a time before anybody could remember, that action became an involuntary habit. What had begun as an act of rational choice had become involuntary, taking place without the individual having any awareness. For Whytt, the ultimate cause of this habitual motion was not straightforwardly material. Instead, he identified an ambiguously immaterial sentient principle, distributed throughout the whole body, as the agent that somehow presided over the acquisition and loss of those habitual motions over which the mind seemed not to have direct control. To use Haller’s terminology, he suggested that the voluntary motions associated with sensibility could, through repetition, be transformed into the involuntary motions associated with irritability – and vice versa.^36^

Here, we can see a clear point of contrast with the embodied, humoral models described by Shapin and Withington. Medical writers had begun to suggest that habits could emerge in the absence of, or perhaps even in opposition to, physical stimuli. In this model, habit could alter the relationship between the mind and the body without obvious changes taking place in the structures of the body, or the fluids passing through it. Instead, a mysterious form of agency associated with the nervous system – but not necessarily identifiable with it – could gradually later the mind’s awareness of its agency over the body.^37^ According to this understanding of habit, the body did not have to undergo material changes in order for its seemingly involuntary dispositions to be transformed. Instead, the force of habit was powerful enough to slowly effect those changes on its own.

## Habit: irritability and human nature in transformation

This vision of habit had implications for the treatment of disease, and for how medics understood the relationship between individuals and society. To grasp precisely what those implications were, we will dwell on Cullen’s discussions of habit, found in the lecture notes taken by his students, along with the published versions of those lectures. As well as devoting long sections of his lectures on the theory of medicine to habit, he frequently brought up habit in the discussions of individual diseases in his pathological lectures, and sometimes in the clinical lectures he gave dealing with hospital patients. Taken together, these discussions reveal that Cullen saw habit as explaining how the body acquired and lost dispositions, and also why certain states of body, including but not limited to diseases, followed repeated patterns that defied physical explanation.^38^

In some discussions of habit, Cullen engaged closely with Haller’s account of sensibility and irritability. In his lectures of 1769 to 1770, for instance, he divided the effects of habit into those on sensibility and those on irritability. Habit, he argued, generally diminishes sensibility, gradually reducing our awareness of sensory perceptions and the actions we undertake. Here, he cited obvious examples such as the diminished pleasure that people receive over time from drinks such as brandy, coffee, tea, and wine. At the same time, he argued that habit usually increases our irritability – that it heightens our involuntary responses.^39^ In his lectures of the following year, Cullen chose a slightly different framework, drawing on the distinction that philosophers such as Joseph Butler had drawn between active and passive habits. Overall, however, the implications were similar. He argued that repeated experiences – passive habits – make us less aware of their effects. We become less sensitive to experience and less sensible of the effort involved with undertaking actions. But he also pointed out that repeated actions – active habits – give us more facility because they make actions easier. But that ease emerged precisely because the mind gradually loses awareness of what it is doing. Habit turned difficult voluntary acts into easy involuntary ones, giving actions over which the mind had once exercised control a kind of automaticity.^40^

In his lectures of 1769–1770, Cullen illustrated these contrasting effects with a striking example that did not appear in any of the published versions that later came out:

Suppose a Man has always lived in a Country, where he has never seen the Face or any other part of a Woman uncovered – this Man woud have some very extraordinary Notions by seeing a Gallery of naked pictures of fine Women. This is a Case of increased Sensibility & woud happen tho’ the State of his Genitals were not in an irritable State – for let him see this often, it w^d^. at last become so familiar as to produce no Manner of Effect – but then let this Man abstain from any venereal Act for a long time - & his Irritability woud become so great, that the smallest Circumstance, as the Squeezing of the Hand or the accidental Blowing aside of an Hankerchief will produce an Excited State of his Genitals.

Here, the male art gallery visitor is overpowered by his first encounter with pictures of naked women. He is aware of having an erotic experience and has an erection in response. As he gets used to visiting galleries, however, the ongoing stimulation gradually recedes from his awareness. At the same time, as Cullen makes clear in his subsequent explanation, the irritability of the man’s genitals increases. Abstaining from sex, moreover, ensures that this man never manages to dissipate any of the accumulated irritability. The result of all this is a new sexual disposition. He may no longer be aroused by paintings of naked women, but he finds himself having erections at the slightest suggestion of sex. Through habit, he loses the capacity to be aroused by paintings of nude women. But at the same time, he gains an involuntary disposition towards sexual arousal.^41^

Elsewhere, Cullen made plain what he had merely implied here – that habit could give the body dispositions that were, for practical purposes, indistinguishable from those given to it by nature, stating in his lectures of 1769–1770 that ‘these Effects [of habit] become established Laws in our System’.^42^ It is worth noting that these implications are already clear in the art gallery scenario. First, the gallery visitor’s initial arousal upon seeing images of naked women is the result of cultural conditioning, and it is this conditioning that makes him so aware of the sexual stimulation that they provoke. Second, it is implied that Cullen and his audience must also have had sexual dispositions contingent on cultural conditions. If eighteenth-century European society made men familiar with images of nude women, it followed from Cullen’s line of argument that those men had been made sexually irritable – far more liable to sexual arousal than men who had not had the same experiences. For Cullen, therefore, social customs and the habits they engendered had the power to alter human nature.

Before proceeding, however, we should note that Cullen used this model of habituation to explain the causes and treatment of a surprisingly wide range of diseases. He regarded habit as significant for conditions including asthma, digestive trouble, fever, gout, haemorrhoids, irregular or suppressed menstruation, and spasmodic diseases such as epilepsy.^43^ What all these conditions had in common was that they involved the recurrence of symptoms after their initial physical cause was no longer present. It was habit, for instance, that explained the way fevers appeared to follow regular patterns, with the symptoms manifesting either daily (quotidian), every third day (tertian), or every fourth day (quartan). His point was that our habitual patterns of life, determined both by the environment and by social customs, imposed long sequences of action on the body. Here, the involuntary association of actions with each other was crucial, making it possible for a single stimulus to activate whole sequences of actions that habit had united.^44^ The result, he suggested, was that fevers were inserted, by force of habit, into these regular, cyclical patterns of associated actions. For Cullen, habit explained the otherwise perplexing tendency of fevers to take on regular patterns regardless of their causes. It was as if fevers were carried along by the momentum of the body’s dispositions, imposing rhythmic regularity on their symptoms.^45^

Physicians generally responded to such maladies not by trying to address their original causes, but rather by correcting the habits that perpetuated them. In 1752 and 1755, for instance, the physician Thomas Simson (1696–1764) wrote to his more famous counterpart John Pringle (1707–1782) about the difficulty of treating spasmodic diseases once they had ‘become habitual’. Confronted by symptoms that seemed ‘as it were periodical […] tho’ the original cause should be removed’, there was no point in trying to cure the original condition. Instead, the solution was to encourage the patient to develop new habits of diet, exercise, and medicine to counteract the ones that had been making them ill.^46^

This is a point that comes through even more clearly in eighteenth-century discussions of strabismus – the condition in which an individual’s eyes do not both point in the same direction. Porterfield and Whytt had both argued that the uniform motion of the eyes was brought about by habit. It stood to reason, therefore, that habit might also explain cases where the eyes did not move in unison. Several authors urged, at least in cases where there was no physical damage to the eyes, that strabismus was simply a bad habit, inculcated by careless parents placing their children in positions that, for instance, left one eye covered, and another uncovered.^47^ As a result, figures such as the philosopher Thomas Reid and the poet, philosopher, and physician Erasmus Darwin (1731–1802) proposed regimes of eye exercise to correct the habit. Reid emphasized the social aspects of his regime too, suggesting that sufferers should encourage friends and family to ‘admonish’ them whenever their eyes were pointing the wrong way.^48^

There were, as I have already suggested, points of continuity between this account of habit and the older humoral one. Both favoured a kind of medicine based on regimen. In turn, this emphasis on regimen belonged to a therapeutic regime based on highly individualised exchanges between physician and patient, with the physician using his expertise to help patients interpret symptoms in relation to an embodied understanding of their own temperaments. But there was also something different afoot. More so than their predecessors, physicians of the late 17th and 18th centuries saw habit as a way of explaining how whole sequences of motion appeared to unfold in the body without the voluntary activity of the mind. Habit provided Cullen and his contemporaries with a way of talking about what we would call unconscious actions and, perhaps more tellingly, the fluctuating boundaries between awareness and unawareness. They were keenly interested in the possibility that choices, whether individual or collective, might through repetition become involuntary, giving rise to a second nature. Yet, this nature was not straightforwardly embodied, emerging as it did through changes in irritability and sensibility that registered neither as obvious changes in the structure of the body, nor in the fluids passing through it. Physicians therefore encouraged patients to imitate the same process of habituation, hoping that the voluntary pursuit of a given regimen might eventually become an involuntary disposition, displacing the pathological one.

## Menstruation as habit

Menstruation can help us to figure out the implications of this model of habit. It brings together the three main themes we have been exploring: the emergence of a vision of habit that was not straightforwardly embodied, the implications of that model for thinking about bodily states and diseases, and the way that model enabled people to explain how environmental and social conditions came to be embedded in the body. It is perhaps surprising, however, that Cullen and other medics of the 18th century regarded menstruation as a product of habit – especially since there has been virtually no discussion of this notion among anglophone historians of menstruation. After all, menstruation is not something that people can simply choose to make a habit of doing. That said, historians such as Sara Read, Jennifer Evans, and Cathy McClive have shown that early modern people recognised menstruation as lying astride the boundary between voluntary and involuntary motion. Medical practitioners, whether men or women, licensed or unlicensed, had long recommended treatments for perceived menstrual irregularities. Women had access to a wide range of medicines intended to provoke menstrual bleeding when it did not take place at the expected time.^49^ In addition, practitioners and patients alike understood that nutrition and way of life affected menstruation, noting that poor people without enough to eat often menstruated less than their wealthier counterparts, especially during winter. People understood that menstruation was amenable to human control and responsive to social and environmental conditions.^50^

In both teaching and practice, Cullen was attentive to the periodicity of menstruation, almost invariably identifying irregular flow as a sign of poor health, to be remedied by some kind of treatment. Since he was not dogmatic about the causes and cures of disease, Cullen gave his students a wide range of possible reasons why menstruation might be impaired. That said, consistent with his belief that the nervous system was the chief agent of motion in the body, he tended to emphasise the possibility that the underlying cause might be a generalized debility of the nerves. In his lectures on menstrual disorders, he therefore tended to discuss treatments designed to restore the nervous system’s responsiveness to the stimuli operating on it. Among the stimulants Cullen recommended were both hot and cold bathing, mineral waters containing steel, electricity, and sexual intercourse – although he seemed to think that it would be difficult to recommend this last treatment to patients without offending them.^51^ We can find evidence of this approach at work in Cullen’s medical correspondence, where he frequently identified menstrual irregularities as symptoms of more generalized nervous disorders. In November 1780, for instance, Cullen wrote to Dr. James Vaughan about an anonymous patient whom he believed to be suffering from a spasmodic nervous disorder that would only be cured once the ‘catamenia have taken their usual flow both in time & quantity’. After discussing a range of ways to bring the patient’s spasms under control, Cullen ventured a course of treatment intended to stimulate the nervous system and thereby promote menstruation. Those treatments included cold bathing and exposure to cold air, as well as riding in a horse-drawn carriage.^52^

Cullen’s tendency to regard menstruation as a nervous phenomenon helps us to grasp how he could characterise menstruation as a habit. In Cullen’s day, the dominant physical explanation for menstruation was the plethora theory, given its most influential telling by the physician John Freind (1675–1728). In his *Emmenologia* of 1703, Freind had argued that fertile women needed to accumulate a store of extra blood in the vessels around the uterus, to nourish any infants they might conceive. Once the accumulation of blood had gone on long enough, the pressure it exerted would force a passage out of the body, emerging as menstrual flow. The pattern would then be repeated, with a new plethora building up until once again the vessels around the uterus could no longer contain the mass of blood.^53^ In the second half of the 18th century, however, physicians increasingly contested this hydraulic account. The problem was that the quantity of blood in the system seemed in fact to make no difference to whether menstruation happened or not.^54^ Cullen pointed out that women continued to menstruate even after they had been bled – an operation that should, according to Freind’s theory, have eliminated the plethora and also the pressure on the uterine vessels. Instead, Cullen suggested that although menstruation was initially brought about by such a plethora, it soon became habitual:

the Nervous System has a share in the Continuance of this Flux, for independent of a considerable Impulse in the Hydraulic System: After the flux has been brought on & repeated a few times it becomes a Nervous Affair or it depends on Habit.^55^

Cullen repeated this point elsewhere, explaining in his lectures on *materia medica* that this habit was so powerful that it made menstruation resistant to medical intervention, with treatments having little effect on its periodical return. Menstruation floated free of its initial cause, depending instead on a habit somehow inculcated into the uterus and the vessels around it.^56^

Cullen was by no means the only figure to link menstruation with habit. The philosopher Denis Diderot (1713–1784) discussed habit several times in his *Élements de Physiologie*, stating that ‘the menstrual flux, at first a need, becomes very periodic by habit, like all the other excretions’.^57^ In his *Système physique et moral de la femme* of 1775, meanwhile, the physician Pierre Roussel (1742–1802) made even stronger claims. Mobilising the vitalist language typical of medics trained at the University of Montpellier, Roussel made it sound as though the uterus itself had a memory, and therefore the capacity to acquire habits: ‘Nature, once relieved by that evacuation, would repeat it at the same time, at first by a confused memory of well-being that she would have received, and then by a kind of habit’.^58^ With its suggestion of embodied memory, this passage is far more confident in its vitalism than anything Cullen said.^59^ Nevertheless, it expresses the same idea. Like Cullen, Roussel identified menstruation as an act that the body undertook regardless of need. There had, they admitted, once been a cause. Over time, however, menstruation became a habit. For Cullen and Roussel, menstruation was not strictly natural. Rather, it belonged to a second nature realised by the force of habit.

Roussel signalled more clearly than Cullen what this notion implied. On the basis of flimsy ethnographic evidence, he echoed Jean-Jacques Rousseau’s (1712–1778) speculations about the effects of civil society on the body, proposing that menstruation may only have come about once humans departed the state of nature and entered the state of society:

All these facts induce us strongly to conjecture that there must have been a time when women were not at all subjected to this uncomfortable tribute, and that the menstrual flux, far from being naturally instituted, is on the contrary an artificial need contracted in the state of society.^60^

Cullen did not himself make such stark claims for the artificiality or contingency of menstruation. But he did suggest that similar processes were involved. In a lecture dealing mainly with convulsions, Cullen argued that the uterus was particularly apt to be affected by changes internal and external to the body, concluding that ‘You will now easily perceive how the Uterus is influenced by Things external & internal to it & how much more considerably by the former’.^61^ In a lecture from the following year, on the pathology of the nervous system, he more bluntly stated that ‘Females […] are more universally irritable than Males’.^62^ For Cullen, women’s bodies – especially their genitals – were more irritable than those of men, and this irritability made them more likely to internalise patterns of action derived from their culture and environment.

We should note, before proceeding, that Cullen saw this greater irritability as a difference of degree rather than of kind. As the example of the art gallery visitor shows, Cullen also associated the male genitalia with irritability. Moreover, like many medics of his day, Cullen still thought that custom and environment could give rise to male bodies with feminine characteristics, and vice versa. In his discussions of haemorrhage and anal haemorrhoids, he gestured to the possibility that some cases might be a form of habitual bleeding analogous to menstruation, arising both in women and in men whose sedentary manner of living made their bodies more feminine.^63^ Nevertheless, both his and Roussel’s remarks fit with two patterns of thought that emerged in the latter part of the 18th century. The first was a tendency to regard women’s bodily and mental development as highly dependent on the state of the genitals. As Sabine Arnaud has shown, this development led many physicians to regard conditions like hysteria, earlier linked to the state of the nerves and fibres throughout the body, as arising from women’s genital disorders.^64^ The second was a widespread belief that women’s bodies – especially with regard to sex and reproduction – were particularly telling indicators of a society’s customs. The suggestion that women’s bodies were particularly irritable provided a biological reason why their bodies appeared more apt than those of men to internalize the customs of a society.

The idea that women’s bodies, more than men’s, could serve as indicators of the state of a society is one with a long and ongoing history. Terry Castle, for instance, has shown how authors of the seventeenth and eighteenth centuries frequently likened women to then recently devised instruments such as barometers and thermometers, seeing female bodies as visibly fluctuating indicators of a society’s customs.^65^ Over the course of the eighteenth century, this notion had an important place in two overlapping fields of intense debate. The first was the debate about the effects of luxurious consumption and sedentary forms of life on human health and morality. The second was the debate about how much human nature could be thought of as unfolding over historical time, changing in relation to cultural and economic conditions. In a sense, the former debate was simply a more contested version of the latter. To worry about the corrupting effects of luxury was, in effect, to worry about what the most recent – and most immediately relevant – phase in the development of civilization was doing to human nature.^66^

Here, a remarkably wide range of thinkers, from physicians and physiologists to historians and philosophers, identified women as bellwethers of society because of the notion that their more delicate bodies somehow absorbed the effects of custom more than those of men. In his widely influential works of medical regimen, for instance, the physician George Cheyne (1672–1743) asserted that effects of leisure and luxury consumption were more palpable in women than men, manifesting in an epidemic of disorders that included hysteria and infertility.^67^ Writing in the 1770s, Diderot used similar ideas about the receptivity of female bodies to suggest that women were ‘so many thermometers of the least vicissitudes of manners and customs.’ Like Cullen, Cheyne and Diderot asserted that women were so receptive to the world around them that they were not fully agents in their own right, almost hosts for the patterns of behaviour imposed on them by society.^68^

There were inconsistencies in this combination of claims about the mutability and fixedness of women’s bodily dispositions. Cullen claimed that women’s bodies were so irritable that they were more likely than men to take on habitual patterns of action. But at the same time he implied, and Diderot stated as a certainty, that this irritability or receptivity was itself fixed. Diderot even claimed that this tendency to receptivity was what made the subjection of women to men natural.^69^ All this is to say that Cullen and others used ideas about habit to deny women the kind of rational, willed agency that they generally found desirable when it came to men. I do not mean to suggest that Cullen was somehow cynical or insincere in his efforts to treat his female patients. The point is simply that his understanding of menstruation depended on gendered ideas about how responsive the body was to habit.

Here, the contrast with contemporary medical and moral approaches to what were then thought of as analogous evacuations in men – haemorrhoids and seminal ejaculation – is illuminating. Simon Richter and Lisa Wynne Smith have both shown that, during the eighteenth century, medical writers came to regard these evacuations in men as the undesirable product of bad habits. Masturbation was of course the most obvious example of such a habit, held up by medics such as Samuel-Auguste Tissot (1728–1797) as one of the defining vices of modern life, resulting in the enfeeblement and feminization of male bodies. Doctors therefore discouraged such habits, insisting that men would obtain ideal states of body and mind by resisting them – by asserting their autonomy over the tyranny of habit and custom. Such advice was consistent, moreover, with Cullen’s ideas about regimen, emphasising as he did the notion that people with male-coded bodies should exercise willed, rational command over the thoughtless impulses of habit. As Rosalie Stott has noted, this ideal was in turn consistent with the Stoic ethic of self-command held up as the highest of moral virtues by contemporary thinkers such as the philosopher Adam Smith.^70^ Crucially, however, medics did not see this kind of autonomy as so desirable in women. Rather, they identified women with a bodily disposition towards the unreasoned formation of habits – a disposition that had the potential to slowly transform them into involuntary vessels of a society’s customs.^71^

Brandy Schillace and others have shown that feminist thinkers of the late 17th and 18th centuries grasped the role that habit and custom played in maintaining this gendering of the body.^72^ In her *A Vindication of the Rights of Woman* (1792), for example, Mary Wollstonecraft made extensive use of a somewhat medicalised vision of habit when making her case that women should have the same political and civil rights as men. Unpromisingly, she made her case by painting an unflattering picture of the women around her, effectively concurring with the notion that their bodies were more delicate than those of men, and more receptive to whatever ideas and experiences were presented to them. Attacking Rousseau, however, she argued that this passive, receptive disposition was far from natural – or at least no more natural than any other disposition arising from social custom. Instead, it was the product of an education that discouraged women from developing strong bodies and preventing them from cultivating their reason. Wollstonecraft thus compared the victims of such an education to dogs that had, over time, come to internalise the signs of their subjection to men:

Considering the length of time that women have been dependent, is it surprising that some of them hug their chains, and fawn like the spaniel? ‘These dogs,’ observes a naturalist, ‘at first kept their ears erect; but custom superseded nature, and a token of fear is become a beauty.’^73^

Simply put, Wollstonecraft held that custom, not nature, enslaved them to men. The very dispositions that male medics and philosophers saw as making women especially receptive to the force of habit were themselves products of habit. And if such dispositions could be made by men subjecting women to bad habits, they could be unmade when women chose to cultivate good ones.^74^

We can therefore draw a direct connection between Cullen’s claims about the causes of menstruation and a widely diffused set of concerns about how social customs might give rise to different varieties of human nature. At the core of these concerns was an understanding that habit could give people agency where they felt as though they had none, but also rob them of agency when they repeatedly exercised it. Wollstonecraft recognised the political stakes latent in such claims about how people related to habit. She saw that men were generally encouraged to mobilise habit in the first sense, using it to gain a kind of mastery over their bodies that would prevent their bodies from simply responding to whatever stimuli the environment and society threw at them. And she also understood that women were generally thought of as relating to habit in the second sense, serving almost as hosts for social norms determined by others. Wollstonecraft did not herself comment directly on medical ideas about the role of habit in menstruation. But her response to contemporary ideas about the effects of habit on women’s bodies helps us to see just how politically fraught Cullen’s claims about menstruation were, turning as they did on inconsistent, gendered claims about how habit alters the body’s patterns of activity.

## Conclusion

Cullen understood that his vision of habit had significant implications for the workings of society. In his lectures of 1769–1770, he argued that habit made possible the regular patterns of action upon which society depended, explaining that ‘If we were exposed merely to external Causes to produce a periodical Return, we shoud be unfit for Society – yet this is corrected by Habit’.^75^ It was habit that prevented people from being dominated by the physical stimuli that would otherwise direct their bodies – whether appetite, excretion, or even menstruation. There was no need for him and his students to contemplate what life would be like if they had to empty their bowels whenever they felt the slightest need, if they had to eat in solitude because hunger made them fill their stomachs at different times, or if women could not predict the timing of their periods. Habit ensured that their bodies would operate in a predictable manner, whether by making the body less responsive to the immediate promptings of physical stimuli or by turning willed actions into processes that no longer required thought or effort to undertake. Somehow, habit could detach the body from the determinations of both will and necessity, imposing periodical regularity on its actions. In turn, this regularity was what made it possible for people to live in harmony with each other.^76^

Cullen and his contemporaries were not simply engaging in philosophical speculation when they made such claims. Rather, they linked their ideas about the workings of society to medical ideas about how to manage the body. And in turn they related those medical ideas to essentially political claims about the roles of male and female bodies, freighting those bodies with strikingly different expectations for agency and passivity, and thereby for freedom and subjection. It is true that Cullen and his contemporaries inherited from early modern humoral medicine rich conceptual and practical resources for managing habit and its tendency to alter the dispositions of mind and body. But the 18th century also witnessed the emergence of a new vision of habit – one in which habit could alter the dispositions of the body in the absence of obvious physical causes. Cullen and Roussel spoke of bodily organs as if they could act intelligently and intentionally, internalizing and then enacting long sequences of action. It became possible to imagine people with receptive minds and bodies as – quite literally – creatures of habit, unfolding patterns of behaviour imposed upon them by the environments and societies in which they lived. We do not yet know, however, what all the consequences of this shift to a neurological understanding of habit were.

Not long after his death, Cullen’s understanding of disease as a matter of temperament would be swept away by successive developments in clinical and laboratory medicine that tended towards the idea that diseases were distinct entities that invaded the body – not imbalances.^77^ It might therefore be tempting to regard Cullen’s ideas about habit as the last iteration in a long series of attempts to explain how temperament might either be maintained or modified. But there is growing evidence to suggest that Cullen’s ideas exerted more influence than might be expected. It was, after all, two of Cullen’s students – the physician and American founding father Benjamin Rush, and the physician Thomas Trotter – who went on to link habit to the mental illness now called alcoholism. Trotter, for instance, argued that alcoholism was fundamentally a disease of habit. It persisted because the mind developed a compulsion for alcohol that floated free on the physical causes actually at work on the body.^78^ It is also likely that Cullen’s conception of habit played a role in the currents of ethnographic and biological thought that thinkers at the turn of the 19th century used to claim that variations in customs and culture led to the formation of biologically distinct racial groups. Bruce Buchan has shown, for instance, that graduates of the medical school at Edinburgh University were crucial to the emergence of colonial ethnography in the early 19th century, sometimes mobilising Cullen’s ideas about the nervous system when attempting to account for racial and cultural difference.^79^ Meanwhile, the literary scholar Shuta Kiba has very recently demonstrated that Erasmus Darwin used a model of habit derived in part from the one promoted by Cullen when he advanced an early model of biological evolution right at the end of the 18th century. There, Darwin argued not only that habitual patterns of action altered the workings of the body, but that those alterations could in some cases become hereditary, leading in turn to the emergence of new and biologically distinctive organisms over time.^80^

Whatever direction future investigations take, it is worth noting that it has only been possible to reveal the political stakes of these ideas by attending to the specific processes and kinds of bodies in which habit was thought to have a role. Although Cullen and his contemporaries saw habit at work in a wide range of conditions, they were particularly interested in those related to the conduct of social life, especially in bodies that were then frequently coded as female – even when found in men. The examples we have encountered, which included the timing of eating and excretion, epilepsy and other spasms, haemorrhoids, menstruation, sexual arousal, and strabismus, were all closely linked in the 18th century to questions about the effects of society on human nature. In addition to menstruation, the examples of epilepsy and strabismus would provide fertile ground for studies examining the social or political stakes of habit as they were related to medicine.^81^ It is true that there was already a tradition of identifying such conditions as the outward signs of inner moral failings, as the long history of physiognomy – the idea that the human facial features are external marks of moral character – bears witness.^82^ What differed in the second half of the 18th century, however, was the way people increasingly came to connect the individual moral stakes of habituation to the idea that human nature itself might be the product of social and cultural conditions, contingent on a society’s economic and political organization. Cullen could imply that a society might make its men sexually irritable through its attitude to nudity in art, while Roussel could argue that menstruation was a product of civilization, unknown to people in the state of nature.

Medical writers saw that people could use habit to liberate themselves from painful medical conditions, gaining mastery over and gradually internalising the cures to some of their afflictions. At the same time, however, they displayed a marked tendency – even in the context of medical writing – to see the use of habit to enhance an individual’s agency over their body as more appropriate to men than women. Cullen and his contemporaries bought into the very widely diffused idea, found in visual art, novels, poetry, and philosophy, that women had less agency than men because they were more likely to be governed by the effects of habit and custom on their minds and bodies. Here, we see in full force something like the vision of habit deployed in more recent years by sociologists such as Bourdieu and Tony Bennett. They point out that habit serves as an instrument of domination, and in their hands the image of habit as the means by which ‘the social order inscribes itself in bodies’ takes on a violent quality. It is surely violence of this kind that Wollstonecraft had in mind when she imagined the women of her day internalising the signs of their subjection in the same manner as dogs cowed by their owners. The medical thought of the late 18th century was shot through with the subjection that habit can inculcate, legitimating gendered fantasies about the effects of the social order on human bodies.

## Footnotes

1

Phil Withington , ‘Addiction, Intoxicants, and the Humoral Body’, The Historical Journal, 65, 1 (2002), esp. 85–88 .

2 See James Kennaway and Rina Knoeff , ‘“The Most Valuable Part of Medicine”: the Six Non Naturals in the Long Eighteenth Century’, in James Kennaway and Rina Knoeff (eds.), Lifestyle and Medicine in the Enlightenment: The Six Non-Naturals in the Long Eighteenth Century (Abingdon: Routledge, 2020), 1–10 .

3

Steven Shapin , ‘Why Was “Custom a Second Nature” in Early Modern Medicine?’, Bulletin of the History of the Medicine, 93, 1 (2019), 1–26 10.1353/bhm.2019.000030956234.

4

Jeffrey Charles Wolf , ‘“Our Master and Father at the Head of Physick”: The Learned Medicine of William Cullen’ (unpublished PhD diss.: University of Edinburgh, 2015), 70–104 .

5 On Cullen’s correspondence with patients, see Wayne Wild , Medicine-by-Post: The Changing Voice of Illness in Eighteenth-Century British Consultation Letters and Literature (Leiden: Brill, 2006), 175–242 17094873. On his work at the Royal Infirmary of Edinburgh, see Guenter B. Risse , New Medical Challenges During the Scottish Enlightenment (Amsterdam: Rodopi, 2005), 19–71, esp. 1–4816212750.

6 See below for detailed discussion and citation of the many places where Cullen discussed habit.

7 John Thomson, *An Account of the Life, Lectures, and Writings of William Cullen, M.D.* Vol. I (Edinburgh and London, 1832). On his friendships with these figures, see 62–74. See also correspondence with Kames, 591–602, and his letter to William Hunter on the death of Hume, 607–609.

8

Joseph Butler , The Analogy of Religion. With a Selection from the Correspondence between Joseph Butler and Samuel Clarke, David McNaughton (ed.) (Oxford: Oxford University Press, 2021), 55–66 . Ultimately, Butler held that habits of virtue were expressions of rational, willed courses of action. Nevertheless, he acknowledged the possibility that ‘actions materially virtuous, which have no sort of difficulty, but are perfectly agreeable to our particular inclinations, may possibly be done only from these particular inclinations, and so may not be any exercise of the principle of virtue, *i.e.* not be virtuous actions at all’ (pp. 62–63). Ideas about habit in medicine and physiology will be discussed below. For a prime example of the role habit could play in the aesthetics of the 18th century, see Henry Home and Lord Kames , ‘Custom and Habit’ in his Elements of Criticism, vol. 1 (Indianapolis: Liberty Fund, 2005), 280–295 . For a summary of the key ideas, see John P. Wright and Kathryn Tabb , ‘Habit and the Association of Ideas in the Scottish Enlightenment’, in Aaron Garrett and James A. Harris (eds.), Scottish Philosophy in the Eighteenth Century. Volume II: Method, Metaphysics, Mind, Language (Oxford: Oxford University Press, 2023), 310–313 . On the scholastic view of habit, see Robert C. Miner , ‘Aquinas on Habitus’, in Tom Sparrow and Adam Hutchinson (eds.), A History of Habit from Aristotle to Bourdieu (Blue Ridge Summit: The Rowman & Littlefield Publishing Group), 67–88 .

9

Kathryn Tabb , ‘Locke on Habituation and the Association of Ideas’, in Jessica Gordon-Roth and Shelley Weinberg (eds.), The Lockean Mind (Abingdon: Routledge), 387–388 ; John Baltes , The Empire of Habit: John Locke, Discipline, and the Origins of Liberalism (Rochester, NY: University of Rochester Press, 2016), 50 ; Thomas Reid , Thomas Reid - Essays on the Active Powers of Man (Edinburgh: Edinburgh University Press, 2010), 88–89 .

10

Roy Porter , ‘Consumption: Disease of the Consumer Society’, in John Brewer and Roy Porter (eds.), Consumption and the World of Goods (London: Routledge, 1993), 62–65 .

11

Robert Wokler , ‘From *L’homme Physique* to *L’homme Moral* and Back: Towards a History of Enlightenment Anthropology’, History of the Human Sciences, 6, 1 (1993), 123–128, 129–132. On William Robertson’s stadial theory of human development, see Karen O’Brien , ‘Between Enlightenment and Stadial History: William Robertson on the History of Europe’, Journal for Eighteenth-Century Studies, 16, 1 (1993), esp. 60–61 . On the use of the treatment of women as an index of progress, see Silvia Sebastani , ‘“Race”, Women and Progress in the Scottish Enlightenment’, in Sarah Knott and Barbara Taylor (eds.), *Women, Gender and Enlightenment* (Houndmills: Palgrave MacMillan, 2005), 83–91 . On the use of climate, environment, and customs to ‘explain’ racial difference, see Roxann Wheeler , The Complexion of Race: Categories of Difference in Eighteenth-Century British Culture (Philadelphia: University of Pennsylvania Press, 2000), 1–38 .

12

John P. Wright , ‘Custom and Habit in Physiology and the Science of Human Nature in the British Enlightenment’, Early Science and Medicine 22 (2017), 203–206 ; Shuta Kiba , ‘Erasmus Darwin and the Biopolitics of the Vital Habit’, Studies in Romanticism 64, 1 (2025), 4–6 .

13 Medical and moral ideas about habit crop up frequently in Sasha Handley’s *Sleep in Early Modern England* (Yale: Yale University Press, 2016). Handley also notes the importance of changing ideas about the nervous system to new ideas about sleep from the late 17th century onwards, an argument that resonates with my own claims about habit.

14 Michael Stolberg briefly addresses the argument made by the 18th-century French physician Pierre Roussel that menstruation might be a product of social organization rather than nature itself. He does not, however, discuss the role of habit in Roussel’s argument. See Michael Stolberg , ‘The Monthly Malady: A History of Premenstrual Suffering’, Medical History 44 (2000), 313 PMC104428610954967.

15 This phrasing comes from Pierre Bourdieu , Pascalian Meditations, Richard Nice (trans.) (Stanford: Stanford University Press, 1997), 141 .

16

Tony Bennett , Habit’s Pathways: Repetition, Power, Conduct (Durham, NC: Duke University Press, 2023), 12 .

17

Tyler Leeds , ‘The Bio-Habitus: Using Pain Science to Reconstruct Bourdieusian Theory’, Sociological Theory 42, 1 (2024), 49–72 .

18 Robert Dobson, ‘Notes from Lectures of William Cullen’, Manchester Medical Manuscripts (henceforth ‘MMM’) 1/1/3, 547–555, Manchester University Library. The dates of the lectures are occasionally given by Dobson in the manuscript. Cf. ‘Lectures on the Institutions of Medicine, by Dr. Cullen’, 1771–1772, MS 3535, ff. 169r-171r, National Library of Scotland; William Cullen, *Lectures on the Materia Medica* (London, 1772), 28–30. This edition was published without Cullen’s authorization, based on student lecture notes. See Wolf, *op. cit.* (note 4), 30, 335–336.

19 Cullen adopted novel theories about the workings of the body and held unorthodox intellectual positions, for instance by repeatedly hinting at the materiality of the mind. That said, his approach to dispensing medical advice was consistent with a long tradition of learned medicine, in which medics used their learning to interpret patients’ own understandings of their bodily states. Jeffrey Charles Wolf therefore characterizes Cullen as ‘not, in the end, a revolutionary but a reformer of learned medicine’. See Wolf, *op. cit.* (note 4), especially 263–264.

20

Robert C. Miner , ‘Aquinas on Habitus’, in Tom Sparrow and Adam Hutchinson (eds.), A History of Habit from Aristotle to Bourdieu (Blue Ridge Summit: The Rowman & Littlefield Publishing Group), 72–73 ; Clare Carlisle , On Habit (Abingdon: Routledge, 2014), 12–13, 97–98.

21 Entries for ‘Custom and Habit’, in *Encyclopædia Britannica*, vol. 5, 3rd ed. (Edinburgh, 1797), 616–623. The entry dealing with the ‘Influence of Custom and Habit on the Mind’ is extracted from the chapter on habit and custom in Kames’s *Elements of Criticism*, and the entry on the ‘Effects of Custom and Habit in the Animal Economy’ is extracted from the section on habit and custom in Cullen’s *Lectures on the Materia Medica.*

22 Cullen, ‘Notes from Lectures of William Cullen’, MMM 1/1/3: 521–523, quotation on 523.

23 In the *Analogy of Religion*, Joseph Butler almost always used the term ‘habit’, although he occasionally used the verb ‘accustom’ to account for habituation. In the *Essays on the Active Powers of Man*, Thomas Reid appears to maintain the Aristotelian distinction between *habitus* and *consuetudo*, reserving the use of the term ‘custom’ for instances of repetition that seemed to him either mindless, or imposed by social custom. Since Reid regarded habit itself as a mindless form of repetition, it is unclear how meaningful this distinction was, however. See Reid, op. cit. (note 8), 90, and John P. Wright , ‘Ideas of Habit and Custom in Early Modern Philosophy’, Journal of the British Society for Phenomenology, 42, 1 (2011), 19 .

24 Shapin, *op. cit.* (note 3), 15–16.

25 Dr E. Smyth to William Cullen regarding Mrs R, 20 March 1784. CUL/1/2/1473, Royal College of Physicians of Edinburgh. Accessed through www.cullenproject.ac.uk, document ID 2418. The term cachexia is interesting here, since its literal meaning in Greek is ‘bad habit’.

26 John Gilchrist to William Cullen regarding Thomas Mutter, 28 May 1780. CUL/1/2/936, Royal College of Physicians of Edinburgh. Accessed through www.cullenproject.ac.uk, document ID 1857.

27 In at least one instance, Cullen attempted to map his own understanding of the temperaments onto the categories of temperament inherited from the broadly humoral medicine of earlier periods. See ‘Extracts from William Cullen’s Lecture Notes’ (ca. 1770), DEP/CUL/3/2/6, f1v, Royal College of Physicians of Edinburgh.

28 William Cullen to Thomas Gillespie regarding John Crawford, 4 June 1776. CUL/1/1/7/46, Royal College of Physicians of Edinburgh. Accessed through www.cullenproject.ac.uk, document ID 3825. Cf. Shapin, *op. cit.* (note 3), 5–6, and Withington, *op. cit.* (note 1) 82.

29 William Cullen, ‘Essay by William Cullen on Custom’, GB 247, Cullen MS 342: 8. University of Glasgow Library. On Cullen’s approach to regimen, see Wolf, *op. cit.* (note 4), 214–255.

30

René Descartes , Principles of Philosophy, Valentine Rodger Miller and Reese P. Miller (trans.) (Dordrecht: Kluver Academic Publishers, 1982), 23–29, 40–41.

31 On God and the soul as the causes of motion, see Descartes, *Principles of Philosophy*, 57. On the causes of motion in the body (and the attribution of most of the motions of the body to physical necessity), see René Descartes , Stephen Gaukroger (ed.) *The World and Other Writings* . Cambridge Texts in the History of Philosophy (Cambridge: Cambridge University Press, 1998), 170–171 . For the key ideas as they related to the interrelationships between mind and body, see John P. Wright , ‘Perrault’s Criticisms of the Cartesian Theory of the Soul’, in Stephen Gaukroger , John Schuster , and John Sutton (eds.), Descartes’ Natural Philosophy (Oxford: Taylor & Francis Group, 2000), esp. 681–682 .

32

Alexander Wragge-Morley , Aesthetic Science: Representing Nature in the Royal Society of London, 1650–1720 (Chicago: University of Chicago Press, 2020), 30–31 .

33 Wright, *op. cit.* (note 31), 688–689. John P. Wright notes that this idea was widely attributed to Stahl by medics of the 18th century. Although habituation played an important role in Stahl’s account of the workings of the body, there seems to be no direct evidence of him repeating Perrault’s exact claim about habit. See Wright, *op. cit.* (note 12), 207. Cf. Elizabeth Haigh, ‘II. Animism, Vitalism, and the Medical University of Montpellier’, *Medical History*, 28, S4 (1984), 28. Thomas Reid also attributed the position to Stahl. See Thomas Reid, ‘Lecture on Pneumatology’, MS2131 8/I/01, p. 10. University of Aberdeen Library. Cullen also identified earlier British medics as ‘Stahlians’. See for instance his discussion of the ophthalmologist William Porterfield in Cullen, ‘Lectures on the Institutions of Medicine’, MS 3535, f. 167r.

34 Peter Hans Reill, *Vitalizing Nature in the Enlightenment* (Chicago: University of Chicago Press, 2005), 130–131.

35 Reill, *op. cit.* (note 34), 128–131; Nima Bassiri , ‘The Brain and the Unconscious Soul in Eighteenth-Century Nervous Physiology’, Journal of the History of Ideas, 74, 3 (2013), 429–434 .

36 Robert Whytt held that an immaterial sensible principle distributed throughout the body brought about all bodily motions, both voluntary and involuntary. See Robert Whytt , An Essay on the Vital and other Involuntary Motions of Animals (Edinburgh, 1751), 268 . See also Nima Bassiri , ‘The Brain and the Unconscious Soul in Eighteenth-Century Nervous Physiology: Robert Whytt’s “Sensorium Commune”’, Journal of the History of Ideas, 74, 3 (2013), 430–431 .

37 Whytt, *op. cit.* (note 36), 119–122; Wright, *op. cit.* (note 12), 200–202.

38 John P. Wright’s discussion of Cullen’s ideas about habit is based on Cullen, ‘Lectures on the Institutions of Medicine’, MS 3535, ff. 97r-195r and William Cullen, *Institutions of Medicine, Part 1, Physiology: For the Use of the Students at the University of Edinburgh* (Edinburgh, 1772), 47–115. To these I have added Cullen, ‘Notes from Lectures of William Cullen’, MMM 1/1/3; 355, 521-557. In addition, there are discussions of habit in MMM 1/1/2, 1/1/4, 1/1/6, 1/1/7, 1/1/8, 1/1/9, 1/1/10, 1/1/11, 1/1/12, and 1/1/13. Habit also comes up in clinical lectures, for example William Cullen, ‘Cl. L. 11^th^ Jan.^ry^ 1774.’, in ‘William Cullen’s clinical lectures, vol. 5, 1773-74’, DEP/CUL/2/1/14, f 1v. Royal College of Physicians of Edinburgh. There are also discussions of custom in various essays and notes by William Cullen in GB 247 Cullen MS 342, 343, 344, 1112, 1113, and 1114. There is also a discussion of habit in Cullen, *Lectures on the Materia Medica*, 21–31, subsequently reproduced in *Encyclopædia Britannica.*

39 Cullen, ‘Notes from Lectures of William Cullen’, MMM 1/1/3: 531-533, 537–541.

40 Cullen, ‘Lectures on the Institutions of Medicine’, MS 3535, ff. 168r-169r. In Cullen, *Lectures on the Materia Medica*, the distinction is less sharply drawn, with more caveats introduced, see pp. 23–27.

41 Cullen, ‘Notes from Lectures of William Cullen’, MMM 1/1/3: 541-543. This example chimes in with 18th- and early 19th-century satires depicting art galleries as settings for titillation and flirting, such as Thomas Rowlandson’s print ‘The Exhibition Stare-Case’, depicting members of the public falling over each other in the pursuit of unclothed flesh.

42 Cullen, ‘Notes from Lectures of William Cullen’, MMM 1/1/3: 521-523. Cf. ‘Lectures on the Institutions of Medicine’, MS 3535: ff. 167r-168r.

43 On asthma, see Cullen, ‘Notes from Lectures of William Cullen’, MMM 1/1/8: 442; digestive trouble, MMM 1/1/13: 2255; fever, MMM 1/1/7: 295-303, MMM 1/1/8: 441-453, and MMM 1/1/9: 731-733; gout, MMM 1/1/10: 1195-1197; haemorrhoids, MMM 1/1/11: 1745-1752; problems with menstruation, MMM 1/1/6: 1601-1605, MMM 1/1/11: 1697-1723; spasms and epilepsy, 2007-2015, 2063.

44 Cullen, ‘Notes from Lectures of William Cullen’, MMM 1/1/7: 293. Cf. ‘Lectures on the Institutions of Medicine’, MS 3535: ff. 167r-168r.

45 Cullen, ‘Notes from Lectures of William Cullen’, MMM 1/1/7: 303. On Cullen’s theory of fevers, and its links to contemporary debates about the effects of climate and custom on the body, see W.F. Bynum , ‘Cullen and the Study of Fevers in Britain, 1760–1820’, Medical History, 25, S1 (1981), 135–147 10.1017/s0025727300070095PMC255736511612144.

46 ‘Medical Annotations Volume 10 by Sir John Pringle’, PRJ 1/10: 65-73. Royal College of Physicians of Edinburgh. The theoretical basis for this approach was new, but the idea of countering a habit with another habit was not. See Withington, *op. cit.* (note 1), 85.

47 The physician James Jurin (1684–1750) had identified habit as the cause of strabismus. Both Buffon and Reid entertained the possibility that strabismus could have habitual causes – especially Reid. However, they both acknowledged that most cases resulted from physical damage to the eye. See Georges-Louis Leclerc , Comte de Buffon , ‘Dissertation sur la cause du strabisme’, Histoire de l’Académie Royale des Sciences Année MDCCXLIII (Paris, 1746), 232 ; Thomas Reid , An Inquiry into the Human Mind, on the Principles of Common Sense, 2nd edn (Edinburgh, 1765), 254 .

48 Reid, *op. cit.* note 47, 248–251; Erasmus Darwin , ‘A New Case in Squinting, by Erasmus Darwin’, Transactions of the Royal Society of London, 68 (1778), 87–88 .

49

Sara Read , Menstruation and the Female Body in Early Modern England (Houndmills: Palgrave Macmillan, 2013), 50, 180 ; Jennifer Evans , ‘“Gentle Purges Corrected with Hot Spices, Whether They Work or Not, Do Vehemently Provoke Venery”: Menstrual Provocation and Procreation in Early Modern England’, Social History of Medicine, 25, 1 (2012), 2–19 ; Cathy McClive , Menstruation and Procreation in Early Modern France (Abingdon: Routledge, 2016), 123–128 ; Michael Stolberg , ‘The Monthly Malady: A History of Premenstrual Suffering’, Medical History, 44 (2000), 315–317 PMC104428610954967.

50 Risse, *op. cit.* (note 5), 292; McClive, *op. cit.* (note 49), 122.

51 Cullen, ‘Notes from Lectures of William Cullen’, MMM 1/1/6: 1599-1619.

52 William Cullen to Dr. James Vaughan, Regarding Anonymous (Patient), 8 November 1780. CUL/1/1/13/104, Royal College of Physicians of Edinburgh. Accessed through www.cullenproject.ac.uk, document ID 1050.

53 Read, *op. cit.* (note 49), 18; Risse, *op. cit.* (note 50), 278; Andrew Shail , ‘Neurology and the Invention of Menstruation’, in Laura Salisbury and Andrew Shail (eds.), Neurology and Modernity: A Cultural History of Nervous Systems, 1800–1950 (London: Palgrave Macmillan, 2010), 61–62 .

54 On the shift to neurological theories of menstruation, see Risse, *op. cit.* (note 50), 280; Stolberg, *op. cit.* (note 49), 310; Shail, *op. cit.* (note 53), 63–67.

55 Cullen, ‘Notes from Lectures of William Cullen’, MMM 1/1/6: 1601-1603.

56 Cullen, ‘Notes from Lectures of William Cullen’, MMM, 1/1/11: 1697-1700; Cullen, *Lectures on the Materia Medica*, 31.

57

Denis Diderot, J Assézat (ed.), Œuvres Complètes de Diderot, vol. 9 (Paris: Garnier Frères, 1875), 398 . ‘Le flux menstrual, besoin d’abord, se périodise beaucoup par l’habitude comme toutes les autres excretions’.

58 Pierre Roussel, *Système Physique et Moral de la Femme* (Paris, 1775), 205. ‘La nature, une fois soulagée par cette excretion, la répéteroit à la même époque, d’abord par un souvenir confus du bien-être qu’elle en auroit reçu, & ensuite par une espece d’habitude’.

59 On Roussel’s vitalism and links to Montpellier, see Elizabeth A. Williams , ‘Physicians, Vitalism, and Gender in the Salon’, Studies in Eighteenth-Century Culture, 29, 1 (2000), 12–13 .

60 Roussel, *op. cit.* (note 58), 197. ‘Tout ces faits nous induisent fortement à conjecturer qu’il a dû exister un temps où les femmes n’étoient point assujetties à ce tribut incommode; & que le flux menstrual, bien loin d’être une institution naturelle, est au contraire un besoin factice contracté dans l’état social’. On Roussel’s engagement with Rousseau, see Kathleen Wellman , ‘Physicians and Philosophes: Physiology and Sexual Morality in the French Enlightenment’, Eighteenth-Century Studies 35, 2 (2002), 267–277 .

61 Cullen, ‘Notes from Lectures of William Cullen’, MMM 1/1/12: 2127.

62 Cullen, ‘Lectures on the Institutions of Medicine’, MS3535 f. 367r.

63 Cullen, ‘Notes from Lectures of William Cullen’, MMM 1/1/11: 1747-1752, esp. 1752; MMM 1/1/12: 2123; ‘Lectures on the Institutions of Medicine’, MS3535 f. 367r. On male menstruation in the 18th century, see Lisa Wynne Smith , ‘The Body Embarrassed? Rethinking the Leaky Male Body in Eighteenth-Century England and France’, Gender and History, 23, 1 (2010), 26–46 .

64

Sabine Arnaud , On Hysteria: The Invention of a Medical Category between 1670 and 1820 (Chicago: University of Chicago Press, 2015), 246–247 . Cullen does this too, for example ‘Notes from Lectures of William Cullen’, MMM 1/1/12: 2125.

65

Terry Castle , ‘The Female Thermometer’, Representations, 17, Special Issue: The Cultural Display of the Body (1987), 1–27 .

66 Wokler, ‘From *l’homme physique* to *l’homme moral* and back’, esp. 42–43; Maxine Berg and Elizabeth Eger , ‘The Rise and Fall of the Luxury Debates’, in Maxine Berg and Elizabeth Eger (eds.), Luxury in the Eighteenth Century: Debates, Desires and Delectable Goods (Houndmills: Palgrave Macmillan, 2003), 15–16, 21 .

67

Anita Guerrini , ‘The Hungry Soul: George Cheyne and the Construction of Femininity’, Eighteenth-Century Studies, 32, 3 (1999), 285 ; George Cheyne , The Natural Method of Cureing the Diseases of the Body and the Disorders of the Mind Depending on the Body (London, 1742), 276–291 .

68 Denis Diderot, J. Assézat and M. Tournot (eds.), *Œuvres Complètes de Diderot*, vol. 2 (Paris: Garnier Frères, 1875), 260. ‘comme autant de thermomètres des moindres vicissitudes des mœurs et des usages’. I first encountered this passage in Castle, *op. cit.* (note 65), 11. See also Laurie Bréban , ‘Women as the Measure of Moral Corruption: Diderot and the Luxury Quarrel’, HAL Open Science (July 8, 2021) <https://hal.science/hal-03010879v3>. On the physiological effects of custom, see Kathleen Wellman , ‘Physicians and Philosophes: Physiology and Sexual Morality in the French Enlightenment’, Eighteenth-Century Studies, 35, 2 (2002), 267–277 .

69

Jenny Mander , ‘No Woman Is an Island: The Female Figure in French Enlightenment Anthropology’, in Sarah Knott and Barbara Taylor (eds.), *Women, Gender and Enlightenment* (Houndmills: Palgrave MacMillan, 2005), 99–101 ; Bréban, *op. cit.* (note 68), 15.

70 Rosalie Stott, ‘Health and Virtue: or, How to Keep out of Harm’s Way. Lectures on Pathology and Therapeutics by William Cullen, *c.* 1770’, *Medical History*, 31, 2 (1987), 126.

71

Simon Richter , ‘Wet-Nursing, Onanism, and the Breast in Eighteenth-Century Germany’, Journal of the History of Sexuality, 7, 1 (1996), 21 11613421; Smith, *op. cit.* (note 63), 31.

72

Brandy Lain Schillace , ‘“Reproducing” Custom: Mechanical Habits and Female Machines in Augustan Women’s Education’, Feminist Formations, 25, 1 (2013), 111–137 .

73

Mary Wollstonecraft , Sylvana Tomaselli (ed.), *A Vindication of the Rights of Men with A Vindication of the Rights of Woman and Hints* . Cambridge Texts in the History of Political Thought (Cambridge: Cambridge University Press, 1995), 161–162 .

74 Wollstonecraft sustains this argument throughout the *Vindication*, but it comes through strongly pp. 161–163. On Wollstonecraft’s engagement with medicine and physiology, see Diana Edelman-Young , ‘Chubby Cheeks and the Bloated Monster: The Politics of Reproduction in Mary Wollstonecraft’s *Vindication*
’, European Romantic Review, 25, 6 (2014), 683–704 , and Kimiyo Ogawa , ‘An Organic Body Politic: Wollstonecraft’s *Historical and Moral View of the Origin and Progress of the French Revolution* and John Brown’s Idea of Health’, in Tristanne Connolly and Steve Clark (eds.), Liberating Medicine, 1720–1835 (London: Pickering and Chatto, 2009), 69–82 .

75 Notes from Lectures of William Cullen, MMM 1/1/3, p. 555.

76 Cullen, ‘Lectures on the Institutions of Medicine’, MS 3535, f. 171r. Also cited in Wright, *op. cit.* (note 12), 206.

77 On the rapid decline of Cullen’s reputation after his death, see Sean Dyde , ‘Cullen, a Cautionary Tale’, Medical History, 59, 2 (2015), 234–240 10.1017/mdh.2015.7PMC440744525766541.

78 Roy Porter notes that there was much continuity between Trotter’s ideas about alcohol dependence, and those of predecessors such as Bernard Mandeville and George Cheyne. See Roy Porter , ‘The Drinking Man’s Disease: The “Pre-History” of Alcoholism in Georgian Britain’, British Journal of Addiction, 80 (1985), 391–393 10.1111/j.1360-0443.1985.tb03010.x3910072. Cf. Withington, *op. cit.* (note 1). But Trotter followed Cullen’s vision of habit when he defined alcoholism as – in part – independent of physical causes. See Thomas Trotter , An Essay, Medical, Philosophical, and Chemical, on Drunkenness, and Its Effects on the Human Body (London, 1804), 172 .

79

Bruce Buchan , ‘Scottish Medical Ethnography: Colonial Travel, Stadial Theory and the Natural History of Race, c. 1770-1805’, Modern Intellectual History, 17, 4 (2020), esp. 927–937 .

80 Kiba, *op. cit.* (note 12), 2–15.

81 Stahl regarded epilepsy as in part a physical manifestation of bad habits. See Francesco Paolo de Ceglia , ‘The Blood, the Worm, the Moon, the Witch: Epilepsy in Georg Ernst Stahl’s Pathological Architecture’, Perspectives on Science, 21, 1 (2004), 1–28 . Epilepsy figured in eighteenth-century debates about the possibility that moral degeneracy could lead to hereditary illnesses. See Sean M. Quinlan , ‘Inheriting Vice, Acquiring Virtue: Hereditary Disease and Moral Hygiene in the Medicine of the French Enlightenment’, Bulletin of the History of Medicine, 80, 4 (2006), 649–675 17242550 10.1353/bhm.2006.0141.

82 The physician James Parsons argued that the repeated expression of a given emotion would gradually give the facial muscles a tendency to rest in that position. See James Parsons ‘Human Physiognomy Explain’d: in the Crounian Lectures on Muscular Motion. For the Year MDCCXLVI’, Philosophical Transactions of the Royal Society, 44, 479 (1747), 43–44 . The artist William Hogarth reproduced these ideas. See Hogarth , The Analysis of Beauty *. Written with a view to fixing the fluctuating* Ideas of TASTE (London, 1753), 129–131 .

